# Meanings attributed to health-related quality of life by caregivers of adolescents with diabetes

**DOI:** 10.1590/0034-7167-2023-0314

**Published:** 2024-06-14

**Authors:** Marina Saraiva de Araújo Pessoa, Elisabeth Luisa Rodrigues Ramalho, Maria Elizabete de Amorim Silva Marinho, Elenice Maria Cecchetti Vaz, Lucila Castanheira Nascimento, Valéria de Cássia Sparapani, Neusa Collet

**Affiliations:** I Universidade Federal da Paraíba. João Pessoa, Paraíba, Brazil; II Faculdade de Goiana. Goiana, Pernambuco, Brazil; III Universidade de São Paulo. Ribeirão Preto, São Paulo, Brazil; IV Universidade Federal de Santa Catarina. Florianópolis, Santa Catarina, Brazil

**Keywords:** Diabetes Mellitus, Type 1, Quality of Life, Family, Adolescent, Caregivers, Diabetes mellitus tipo 1, Calidad de Vida, Família, Adolescente, Cuidadores

## Abstract

**Objective:**

to interpret the meanings attributed to the health-related quality of life by caregivers of adolescents with type 1 diabetes mellitus.

**Methods:**

qualitative, descriptive-exploratory study of 14 caregivers of adolescents with diabetes developed at the reference outpatient clinic for endocrine diseases in a city in the state of Paraíba. Interviews were performed between May and September 2021. Inductive thematic analysis of the empirical material, and its interpretation in light of the concepts of health-related quality of life and family functioning were performed.

**Results:**

the meanings attributed by caregivers to the health-related quality of life of adolescents converged on the feeling of being healthy, healthy eating, satisfactory family income, family involvement in care and effective access to the care network.

**Final Considerations:**

knowledge of these meanings enables health professionals to develop strategies that meet the unique demands of caregivers experiencing this diagnosis.

## RESUMEN

**Objetivo:**

interpretar los significados atribuídos a la calidad de vida relacionada con la salud por cuidadores de adolescentes con diabetes mellitus tipo 1.

**Métodos:**

estudio cualitativo, descriptivo-exploratorio con 14 cuidadores de adolescentes con diabetes desarrollado en el ambulatorio de referencia para enfermedades endocrinas de una ciudad del estado de Paraíba. Las entrevistas se realizaron entre mayo y septiembre de 2021. Se realizó un análisis temático inductivo del material empírico y su interpretación a la luz de los conceptos de calidad de vida relacionada con la salud y funcionamiento familiar.

**Resultados:**

los significados atribuídos por los cuidadores a la calidad de vida relacionada con la salud de los adolescentes convergieron en el sentimiento de estar sano, alimentación saludable, ingreso familiar satisfactorio, involucramiento familiar en el cuidado y acceso efectivo a la red de cuidados.

**Consideraciones Finales:**

el conocimiento de estos significados permite a los profesionales de la salud desarrollar estrategias que atiendan las demandas únicas de los cuidadores que experimentan este diagnóstico.

## INTRODUCTION

The number of children and adolescents with type 1 diabetes mellitus (DM1) grows annually, and a large part of this population is in Brazil, which ranks third in the age group from zero to 19 years with the diagnosis of the disease in the world^(1)^. Few countries in the world have data on the disease incidence in children under 19 years of age, and it is likely that these are underestimated^(2)^.

When experiencing this complex disease that requires continuous care management, many family members feel unprepared, distressed and guilty. Initially, they present difficulties in accepting the diagnosis, mainly in relation to its chronicity^(3,4)^.

Caregivers are often part of the family nucleus of the adolescent with DM1 and need to be included in the care plan drawn up for thefamily, since the health-related quality of life (HRQoL) of caregivers is also affected. Most of the time, mothers are the main caregivers and experience biological, psychological and social implications triggered by the demands of daily care for adolescents with DM1. Therefore, it is necessary to strengthen the support network for these families, seeking to bring them into the focus of healthcare^(5)^, facilitating the experience with DM1 and improving their HRQoL.

The “health-related quality of life” concept can contribute to understand the meanings of the experience lived by families of adolescents with DM1, as it is a subjective, multidimensional and dynamic construction, associated with individual, social and environmental factors that provide the well-being of the individual. The subjective aspect of this concept seeks to understand the person’s perception of their health and their life situation, based on howthe disease interferes in an individual’s life^(6,7)^. Understanding the meanings attributed to HRQoL by caregivers of adolescents with DM1 makes it possible to understand which circumstances influence their HRQoL, allowing that family members provide interventions based on their needs.

The family and caregivers of these adolescents are seen as supportive in the context of the disease and in most cases, they suffer the greatest care burden, which directly interferes in the biopsychosocial context^(4)^. Given the implications for those living with adolescents with diabetes in the family, the following question was raised: What are the meanings attributed by the caregivers of adolescents with DM1 regarding their HRQoL?

## OBJECTIVE

To interpret the meanings attributed to health-related quality of life by caregivers of adolescents with type 1 diabetes mellitus.

## METHODS

### Ethical aspects

The ethical precepts of the National Health Council were followed in this study and it was evaluated by the Research

Ethics Committee. After each participant was explained about the study and understood it, the Informed Consent Form (IC) was presented for signature and consent to enter the study. In remote interviews, the IC was sent through a Google Forms’ link using the WhatsApp application. Participants were represented by the letter I for interviewee, followed by an ordinal numeral representing the order in which interviews were carried out (I1, I2, ..., I14) to guarantee confidentiality.

### Theoretical-methodological framework

The Calgary Family Intervention Model (CFIM)^(8)^ was adopted, as it allows understanding and planning actions aimed at families. The aim of this model is to seek solutions to minimize the emotional, physical and spiritual harm caused by the disease in thefamily^(8)^.

### Type of study

Qualitative, descriptive-exploratory study, guided by the recommendations of the Consolidated Criteria for Reporting Qualitative Research (COREQ)^(9)^. The HRQoL was investigated from a qualitative perspective due to the subjective dimensions of this construct, which relate the health and illness process with the repercussions on people’s lives, including physical, mental, social and occupational aspects^(10)^. Additionally, the HRQoLconstruct has been explored in the literature through measurement instruments that certainly contribute to its understanding, but predetermine its assessment based on a set of items and dimensions. The qualitative perspective of this construct expands the apprehension of HRQoL, giving visibility to the experiences of caregivers.

### Study setting

The study was carried out at the Endocrinology Outpatient Clinic of a public hospital in a large city in the state of Paraíba, Northeast Region of Brazil. The service is a reference for the treatment of DM1 in the municipality studied and in other municipalities in the state that are covered by the institution.

### Data collection

The main caregivers of adolescents with DM1 participated in the study. The person in the family who routinely managed DM1 in partnership with the adolescent was considered the main caregiver. This identification was based on the narrative of the family member approached by the main researcher in the first contact to explain the objective of the study and assess the selection criteria for participation in the study.

Steps were taken to recruit participants. Face-to-face contact with caregivers was established on the day of the adolescents’ outpatient follow-up appointment, as well as via WhatsApp later on. In the latter case, the invitation to participate was made individually to each caregiver through contacts of the WhatsApp group of caregivers of children and adolescents with DM1 treated at the outpatient clinic. The main researcher was added to this group after prior contact with the group administrators, who were also followed up at the outpatient clinic under study. Before entering the collection field, a nurse (first author) was trained by other team members with extensive experience in research. In the qualitative collection of data, the approaches and data collection were performed by the nurse who had been previously trained.

The inclusion criteria adopted were: being the main caregiver of an adolescent with DM1 for at least six months, as this is a period in which the caregiver has had experience with the treatment; and being the person who performs the daily management of DM1 with the adolescent. The exclusion criteria were: caregivers of adolescents who stopped the monitoring more than six months earlier, despite having some record of care at the outpatient clinic; and those without the conditions and/or technological skills for the remote interview.

The interviews were carried out between May and September 2021; ten remotely and four in person. The sufficiency criterion supported the closure of the interviews, since the set of data collected made it possible to understand the object of study^(11)^.

### Data analysis

Inductive thematic analysis (ITA) procedures were used in the empirical material^(12)^, which was interpreted in light of the concept of HRQoL^(10)^ and family functioning, according to CFIM assumptions^(8)^. In this model, the category of family functioning is divided into instrumental and expressive. The instrumental is focused on Activities of Daily Living, for example, administering insulin, carrying out capillary blood glucose measurements, among other aspects of the care for adolescents with DM1. Expressive family functioning involves nine other categories: emotional communication, verbal communication, non-verbal communication, circular communication, problem solving, roles, influence and power, beliefs, alliances and unions^(8)^.

In the first phase of the analysis, the research team carried out repeated readings of the interviews transcribed in full, so researchers could become familiar with the data set and understand its scope^(12)^. This set of data constituted the corpus of the analysis. In the second phase, the process of generating initial codes began, enabling the initial characterization of data. To this end, significant extracts were highlighted in different colors using the Microsoft Office Word®. In the third phase, the codes were grouped according to the affinity and relationships established between them to generate themes^(12)^.

In the fourth phase, after refinement, the descriptive themes were established. In the fifth phase, an analytical theme was constructed based on the synthesis of descriptive themes and coordinated with the functional aspects of the CFIM. In the last phase, the final reflective analysis was performed based on the recognition that the extracts included the representation of the object of study^(12)^, as well as on the articulation between the themes with the literature and the researchers’ interpretation.

A set of procedures guaranteed the rigor of this study: a sufficient number of interviews was performed with participants, which allowed deepening into aspects that expanded the understanding of the object of study; data collection methods and processes were described in detail; data on the characterization of caregivers were presented to understand the context of the participants’ experience; a thorough analysis of data was conducted with a presentation of the interpretation of results illustrated with excerpts extracted from participants’ reports, and coordinated with the literature and functional aspects of the CFIM theoretical framework^(8)^.

## RESULTS

Fourteen caregivers of adolescents with DM1 participated in the study; 13 mothers and one father, aged between 34 and 56 years. The majority declared themselves to be married and having completed secondary school. The most frequently reported occupations were farming and homeworker. The time as a caregiver for an adolescent with DM1 varied between three and 15 years. Note that only one out of the 14 participants was male.

### Initial repercussions of the disease for the caregiver and family of the adolescent with DM1

Upon receiving their child’s DM1 diagnosis, caregivers experience a feeling of guilt, because of their belief of having responsibility for this outcome.

*I felt like I was guilty of something; I thought she’d gotten like this because of me.* (I10)

The way they received confirmation of their child’s diagnosis, not always with comprehensible communication, influenced the initial confrontation. Although bewildered and without understanding what their lives would be like from that moment on, they took on the role of caregivers and tried to be strong, so as to minimize the impact of the disease on their child.

*She* [physician] *arrived and said that he* [son] *was diabetic and that diabetes was a disease for which there was no cure. He already started crying. As a mother, my world collapsed there. He looked at me and asked: “Mommy, am I going to die?” I said: “No, you’re not, son”. My world fell apart, but on the other hand, I gave him the strength to move forward.* (I2)

The burden of care resulting from the initial treatment of DM1 can create stress for the caregiver and the entire family. In general, because they did not know the disease and the necessary care, the initial reactions were anguish, fear and denial. Proactive actions can be immobilized by the creation of lines of escape, making it difficult to learn and cope with daily care at home.

*We were very distressed, because no one knew how to take care of it. My husband was injecting* [insulin]*, but one day he wasn’t home and I had to do it, nervous, shaking. I was scared, I just cried and cried, I said I wouldn’t be able to prick the finger, that I wouldn’t be able to inject* [insulin]. (I9)

### Quality of life: new meanings based on reflections between the past and the present

Experiencing a chronic disease such as DM1 in the family influenced the caregivers’ perception of their HRQoL, referring its meaning to factors related to health, healthy eating, leisure, physical activity, employment, family union, the absence of diseases, ease of access to materials and supplies, tranquility and peace. For participants, quality of life was:

[...] *we can make an appointment to see a physician and have more space to go for walks, go to the gym. Having a good diet, eating healthy, that’s quality of life.* (I1)

[...] *having your family, having food, being healthy, going out a little, having fun. It’s about having health, peace, tranquility.* [...] *it’s people having their job, earning their money. Because if you have your job, you won’t humiliate yourself by asking anyone, you’ll be able to feed your children well, you’ll live more peacefully.* (I12)

With the diagnosis of DM1, family interactions and the roles to be played were reorganized. Caregivers became more attentive and concerned about their children and felt less free comparing to the period before the diagnosis. Health-related quality of life was considered better when there was no chronic illness in the family.

*I felt freer, less worried. Because, you know, if he didn’t have this disease today, for example, and I’m going out, I wouldn’t worry as much as I worry about leaving him alone.* (I13)

The caregivers renounced their wishes, ceasing to carry out activities that were part of their daily lives, such as going to places where there would be sweets, so their children would not feel excluded.

*I’ve already stopped going to some places, for example, birthday parties that have sweet things, delicious things, I preferred not to go because of her, because going with her and she’d just watch people eat, it got annoying.* (I5)

DM1 also changed the caregivers’ sleep routine due to concerns about managing the disease or the demands of other children. The possibility of nocturnal hypoglycemia or other complications triggered interruptions in sleep and rest.

*It had a huge impact on my sleep, I had to get up several times at night, because I got worried that the blood sugar had dropped, if she* [daughter] *was breathing, I kept thinking a thousand things. The next day, I woke up very tired because I hadn’t slept at night.* (I6)

The working lives of caregivers were also compromised due to the new roles to be played in the care for their children. Some had to leave their jobs due to the daily demands imposed by therapy, making them feel restricted given the attention they had to offer their children.

*Before his diabetes I’d go out, sold perfume, always doing something, and after his diabetes it was something that was more limiting for me; the attention was on him, especially because at the time* [of diagnosis] *he was so small, he depended on me to inject insulin.* (I12)

### Carrying on alone or with support: the support that makes a difference in everyday life

It was difficult to build new alliances to share care. However, some caregivers reported receiving certain forms of support: from their spouses in performing care related to DM1 and sharing household obligations; from their other children, who were attentive to the complications that their siblings might present; from other mothers, through a social network group, receiving informational support related to their daily lives with DM1; from people of their religion/spirituality, with important support for caregivers to “move forward”; and the outpatient reference, where professionals offered strong bonds of support and trust in the care provided from the beginning of the diagnosis.

*My husband helps me a lot, when necessary, he does the tests, injects insulin, and also cares for his* [son with DM1] *diet. Sometimes, I have to visit the physician with one of the children, he stays at home and does everything, both in terms of household chores and with them* [children]*. My daughter, who doesn’t have DM1, pays a lot of attention, sometimes they are feeling something and she is very attentive to her brothers* [both with DM1]*, if they need something, if they are feeling unwell. She is a great support.* (I4)

*I held on to God, I asked Him to help me. I would have to be strong to help, because she was the one going through the difficult situation* [daughter with DM1] *and I am sure He heard me, from then on, he put peace in my heart and understanding so I’d be able to move on with my life.* (I6)

*It helps a lot* [WhatsApp group]*, answers questions, as I live in a distant town and when some information was missing, I couldn’t call C.* [medicine dispensing location] *to find out if there was insulin, something, the girls of the group would always do this. It’s always like that, one person helping the other or with a word.* (I12)

*Thank God I am grateful, because they* [outpatient clinic professionals] *are very patient, they are always willing to do what they can, to give advice, I am satisfied. I have O.’s contact details, who is a nurse there, and whenever I need any information, I send her a message, and she gets back to me with the answer.* (I14)

An important type of alliance in this process was that established between the adolescents’ friends with the caregivers and/ or with adolescents themselves, as friends were considered as strong bonds in the social support network. Friends’ interest in DM1 encouraged adolescents with diabetes to follow treatment correctly, take care of their health and be aware of possible acute complications.

*They* [the adolescent’s friends] *became more attentive, they called her to see how she was, they called me to ask what they could do. I explained them, I said that the day she felt sick they would have to call me, so I could go there with the medication. It’s good to share it* [with her friends]*, because this disease was new to everyone* (I3).

But not all participants received family support in managing the disease at home, making it difficult to solve problems. Justifications such as fear of injecting insulin and lack of courage to perform care, made caregivers feel disappointed or alone in dealing with the diagnosis and therapy, weakening intra-family relationships and consequently, the alliances and unions between their members. People at work and professionals at the adolescent’s school were also indifferent to the difficult time experienced by the caregivers and did not offer support.

*He* [the teenager’s father] *says he doesn’t inject insulin because he’s afraid. The brother doesn’t get involved because he doesn’t have the courage, the care is more with me. At first, I was a little disappointed, but now I don’t care. I say: “thanks God that gave me strength”.* (I2)

*I haven’t received support in my workplace, from anybody, I think they pretended they didn’t understand or didn’t want to understand. At school it was no different, it was very complicated, because teachers had no knowledge about how to act, what to do.* (I6)

### New impasses and resignifications meanings for coping with diabetes

The length of experience in managing DM1 positively a negatively influenced the caregivers’ perception of HRQoL at t time of the interview. Those who adapted positively had a bett perception of HRQoL, while those who had more difficulties dealing with the disease reported a worse perception of HRQo The first case was related to the family’s greater involvement care, for example, with the search for a healthy life for all membe In the second case, the feeling of lack of health, reduced incom and a deficit in sharing responsibilities, led to a worse perceptio

*I think it’s not that good* [HRQoL] *because there’s a lack understanding, a lack of dialogue, I notice I’ve already tried, I talked and it’s getting harder and harder. I’m trying to impro my quality of life, doing what’s best for me, my quality of sleep. I’m sleeping better because I didn’t sleep, now I’m sleeping the whole night* [...]. (I10)

*I have the responsibility of paying for water, electricity, telephone, internet so they can study, and all of this is me alone, thank God there is help from B.* [government benefit]*, which also helps. But to tell you that a person has a quality of life without a job and earning money to at least eat well...no, I don’t consider it quality of life.* (I12)

For the future, caregivers demonstrated expectations of positive changes in their health when they took care of their well-being. As for care management, they believed in the possibility of oral therapy, instead of injectable insulin, due to the discomfort related to pain during injections.

*Lately, I’ve been walking, eating better, trying to lead a healthy life, because I was so overweight, I think I’ve improved a lot.* (I10)

*I wish it was a pill* [DM1 treatment]*, not insulin so I wouldn’t be pricking. It bothers me, I think it hurts.* (I7)

They also would like there were people to share the care of their child with DM1 on a daily basis, thereby strengthening alliances and allowing them to free themselves a little from the role of caregiver.

*I would like to have that person present to help, but it’s me or him* [son with DM1]*, if I have to go out, he* [DM1 son] *takes care of himself, injects* [insulin] *and eats what I prepared.* (I9)

### Health-related quality of life trail for caregivers of adolescents with DM1

**Figure 1:**
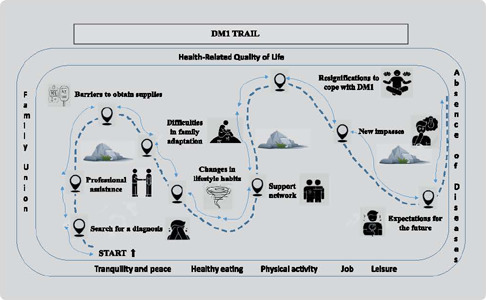
Health-related quality of life trail for caregivers of adolescents with DM1, 2022

Figure 1 represents the analytical theme “Health-related quality of life trail for caregivers of adolescents with DM1: transitions in care management and mobilization to restore family homeo-stasis”. The term “trail” was used to metaphorically represent the paths taken in the search for solving problems and the difficulties faced by caregivers in rebuilding family homeostasis and HRQoL. However, each caregiver presented singularities in the way they saw their own future and that of the family and the adolescent with DM1, influenced by the way in which the instrumental and expressive dimensions of family functioning were established before, during and after this diagnosis.

The caregivers faced different daily experiences following the diagnosis of a child with DM1. They noticed a decrease in HRQoL given the great demand for care, restrictions and negative feelings and uncertainties that became part of family life.

After a significant period of experience, the caregivers’ perception of HRQoL was influenced by their knowledge of DM1, family relationships, support network and adolescent adherence and commitment to treatment. This perception was greater in families who shared care among themselves and were monitored by the outpatient reference service. In this way, the caregivers constantly followed a retroactive and endless trail.

The way they faced the disease was influenced by family relationships that pre-dated DM1; barriers related to necessary supplies; local healthcare they received; difficulties in family adaptation after the diagnosis and new impasses that could arise over time. Family interactions and the mobilization of all family members were important for mothers/caregivers feeling embraced and less overwhelmed by what they were experiencing. Family unity in the performance of different roles directly interfered with the caregivers’ perception of HRQoL. When the family remained or became united, there was a greater perception of tranquility and peace on the part of the caregiver, as well as availability of time to perform physical activity and have a formal job and leisure time.

## DISCUSSION

Among the 14 caregivers of adolescents with DM1 participating in this study, 13 were mothers and only one was a father. This allows considerations about family expressive functioning in terms of beliefs. Women assume this role of care in the family, as they are considered responsible for the health of their children^(13)^. Gender differences place mothers more frequently in the role of caregiver, which can negatively impact family functioning^(8)^.

Caregivers had difficult experiences when faced with the diagnosis of DM1. Accepting a child with a chronic illness is a continuous process in which initial feelings can be transformed into conformity as adolescents follow the treatment^(14)^. The ways in which mothers and fathers are embraced by professionals and information is shared with them affect the understanding of the disease and can minimize or maximize the initial suffering of caregivers. It is important that professionals are empathetic and sensitive, seeking to understand the psychological and emotional impact suffered by caregivers and adolescents. A sensitive and humanized approach enables the formation of close bonds between professionals and the family, as well as the establishment of more effective care plans^(15)^.

Caregivers need partnership and help from other family members to lessen anxieties and worries. This way, they can feel safe sharing their fears^(16)^. Reluctance to learn care practices may reflect on the way children face the diagnosis. Therefore, health professionals also play a fundamental role in care guidance^(17)^.

Caring for an adolescent with DM1 involves economic, affective, social, behavioral, psychological and professional factors. Therefore, it is a dynamic and complex process that also depends on the personal qualities and skills of the caregiver^(5)^. The aspects brought by caregivers about their HRQoL are in line with their concept, as changes in a child’s health status influence the lifestyle and social satisfaction of the family, which are decisive in coping with the disease^(10)^. Additionally, the bonds between family members or emotional ties before the diagnosis of DM1 exert influence on family unity in the face of the health-disease process. When the family has close ties before the diagnosis, the participation of all family members in care is easier^(8)^.

In general, the study indicates that the quality of life of caregivers of children and adolescents with a chronic illness was negatively influenced by the number of children with this condition, the caregiver’s use of medication and financial limitations. The protective factors, which favor quality of life, were having the own home and the support received^(18)^.

Negative initial experiences were also identified in a study that considered the period immediately after diagnosis as a moment of disruption in the lives of caregivers. This disruption was related to the impairment of domestic, social and professional activities, highlighting difficulties in solving problems in the family system. Difficulties in accepting changes were also evident in the new imposed routine. Therefore, the support of a constantly accessible multidisciplinary team is a priority to minimize concerns and anxieties that negatively affect HRQoL^(19)^.

DM1 within the family and the adversities resulting from the disease create new relationships between family members. With support of the CFIM, such relationships can be positive, when they allow sharing the care for the adolescent with other members, or negative, when the mother assumes all responsibilities^(8)^. Relationships are influenced by the age of the child/adolescent; the paternal emotional balance in the face of the diagnosis, commonly greater compared to that of mothers, since it is more difficult for fathers to express affection in emotional communication; and by the often stronger bond between mother and child, unlike the father, who can be excluded from care by caregivers when there is no psychological support and guidance^(20)^.

Given the reality presented, it is understood that the meanings attributed to HRQoL by caregivers are also related to the functioning of each family. In situations where the adolescent requires a great demand for care, especially at the beginning of the diagnosis, caregivers may feel exhausted or frustrated by the failure to perform their individual activities. Therefore, caregivers’ HRQoL meanings are influenced both by the way they understand family functioning and by the way care is shared (or not) among members. Establishing new routines related to the instrumental aspects of the CFIM as an active behavioral response to the disease with the participation of all family members is an important mechanism for maintaining a sense of family normality^(8)^.

In the expressive dimension of family functioning, caregivers assume the central role in care and begin to have their sleep interrupted to meet the demands of their child with DM1. Concerns about acute complications triggered during the child’s sleep generate anxiety and stress, impacting their rest. Impaired sleep has psychosocial implications for the lives of caregivers, which is why evaluating family and adolescent sleep is essential. Nighttime management can be better adapted, establishing the sharing of nighttime care by different family members, when possible^(21)^.

Being part of working life contributes to a better HRQoL of caregivers. However, for them, this option is eliminated by the daily demands of managing child care. Thus, family income is often compromised, which, combined with low education, leads the family to a vulnerable situation. Compromising income can double the chances of low QoL for caregivers of a child or adolescent with a chronic illness^(18)^. Furthermore, all family members become financially dependent on a single member – in this case, the father. This fact is negatively associated with the roles in sharing care.

It is often observed that the husband’s income is negatively related to the division of roles in the family, while the wife’s education is positively related. According to the CFIM, the father figure is strongly associated with socioeconomic aspects of the family and less actively with its functioning, unlike women. Fathers have more rigid standards and difficulty in expressing emotions, which interferes with the family’s emotional communication^(8)^.

The support network is one of the most important positive influencers for the family and adolescents. The family itself, the social network and religion can contribute to overcoming the difficulties and overload faced by caregivers after the diagnosis. The family helps with affection, financial support and actions in everyday life; social media groups help with the lack of supplies and by exchanging experiences; and religion, in emotional aspects of comfort^(8,22)^. Religious or spiritual beliefs are important for the functional aspects of the family, as they are associated with its values and culture. When a member suffers a health problem, restricted beliefs can make it difficult to cope with the disease and generate suffering due to a lack of perspective^(8)^.

The diabetes education team at the reference outpatient service becomes an important source of support for the family, especially during adaptation to the demands of the disease. Professionals give caregivers greater confidence and security in the guidance they receive, reducing barriers between care and families, improving disease control based on motivations brought by adolescents. Accessibility to a team of professionals who seek to understand the family reality and plan care according to their needs provides greater satisfaction with the healthcare received^(23)^. The CFIM is an important tool that can be used by professionals to macro-evaluate families, as planning interventions aimed at problem solving can positively interfere with family functioning.

The support from adolescents’ friends brings security to mothers, especially at school or in moments of social interaction. For adolescents, it can be fundamental for acceptance of the disease and adherence to treatment when friends are concerned about their well-being and interested in learning about diabetes, and can help them in cases of identifying and correcting glycemic changes^(24)^.

The social support offered by peers also becomes important in the coping process, through the exchange of experiences and information, and by facilitating the acquisition of materials and supplies for therapy, for example. Furthermore, it contributes to increase esteem through established relationships. Such actions strengthen adaptive behaviors and self-confidence and provide visibility for caregivers^(25)^.

Corroborating the findings, the lack of support from the adolescent’s school was also evident in a study conducted in Saudi Arabia, which sought to investigate school safety and the differences in diabetes treatment between public and private schools. Parents had a poor perception of the care provided, as children and adolescents were responsible for the treatment alone, especially in public schools^(26)^. In a Brazilian study, the school team presented low knowledge and difficulties in understanding the needs of children and adolescents with DM1, highlighting the need for public policies focused on education on the appropriate management of the disease at schools^(27)^, which is fundamental, as these are individuals in a school dyad.

Gender relations represented by mothers assuming responsibilities for care mark the definition of roles, and the overload of tasks often leads to mental and physical health losses for caregivers^(8,28)^, with a decrease of HRQoL compared to the general population. On the other hand, they use resilience to deal with the overload of care, with a different view of what they are experiencing and a focus on problem solving^(8)^. These coping mechanisms help to develop skills to withstand the difficulties imposed by DM1, as well as to focus on their ability to respond positively to their child’s daily demands.

Although children with DM1 grow up and begin to develop autonomy in self-care, the burden of worries remains high, harming the health of caregivers due to physical and mental stress that is strengthened by exhausting family relationships. A care plan involving the family in the process and aimed at reducing conflicts tends to improve caregivers’ perception of their health^(29)^.

A Brazilian study on the QoL of caregivers of children and adolescents with DM1 showed low scores in the physical, psychological and environmental domains. These aspects reflect low positive feelings, self-esteem, body image, socioeconomic factors and sleep, leading to losses in health conditions^(30)^. Another study with Lebanese families in which most mothers did not have higher education and were housewives, showed that the perception of family HRQoL was directly linked to functional problems in the family group^(31)^. It is important to know how relationships were established pre-diagnosis of DM1 and the family culture, in addition to current family relationships, as these factors interfere with the family’s better or worse adaptation to the disease^(8)^.

The HRQoL of caregivers in this study is related to the changes in social and financial aspects triggered by the care for the adolescent within the family. The experiences they had after a period of diagnosis improved their view of DM1, even though their daily lives were still permeated by concerns that often lead to disagreements within the family. The HRQoL of these caregivers is influenced by several determinants, and family involvement is one of the most important. However, this depended on how these bonds were formed between the members and whether or not each member shared responsibility for the care of the adolescent with DM1.

Caregivers have optimistic expectations, in which they project future improvements for their health conditions and those of their children, as well as healing beliefs, showing the need to reduce negative feelings^(32)^, which can lead to future frustrations. Despite the difficulties faced by caregivers in their daily lives of dealing with DM1, expectations can motivate them to take care of their own well-being.

Figure 1 “Health-related quality of life trail for caregivers of adolescents with DM1: transitions in care management and mobilization to restore family homeostasis” represents the daily lives of the families interviewed in this study. With the children’s diagnosis of DM1, there were changes in the instrumental and expressive aspects of family functioning, especially that of the caregiver. Families that are more flexible in adapting and share the responsibilities of care for the adolescent and their concerns tend to form a positive coalition. Monitoring these families has become essential, as health professionals can help them find their homeostasis, favoring family functioning.

### Study limitations

The participation of only one caregiver father stands out as a limitation of the study, making it impossible to differentiate and deepen the singularities of the father figure in the care for a child with DM1.

### Contributions to the health sector

The contribution of this study for practice regards the subjective understanding of the HRQoL of caregivers and families, enabling the creation and/or improvement of public policies and professional practices to guarantee unique, comprehensive and effective care and improve the HRQoL of caregivers, families and adolescents with DM1. In this way, they will be strengthened and helped in finding their own solutions in the face of everyday adversities.

## FINAL CONSIDERATIONS

The perception of the caregivers interviewed about the meanings attributed to HRQoL is related to the perception of health, well-being and avoidance of illness; access to healthy food for the whole family; moments of collective fun; unrestricted physical exercise; formal employment as a secure source of income; living with serenity; maintaining family unity; and obtaining the necessary care supplies without bureaucracy, with access to the health care network. In general, HRQoL was considered worse by caregivers at the beginning of their children’s DM1 diagnosis, but improved over time with the families’ experience. The vast majority still does not consider it satisfactory, despite countless experiences related to the disease.

The optimistic perception of family functioning was present in families with a support networkformed by spouses and other children who helped with the demands of DM1 care and domestic activities; by adolescents themselves committed to their treatment; by the children’s friends, encouraging them to follow the treatment; by other families experiencing the same condition and by social media, which provided information and donations of supplies; and for the embracement offered by outpatient professionals. With the support given, negative changes related to DM1 had less impact on caregivers’ lives.
