# Supplementary material for: Meanings attributed to health-related quality of life by caregivers of adolescents with diabetes
Source: Rev Bras Enferm. 2024 Jun 14;77(2):e20230314. doi: 10.1590/0034-7167-2023-0314 (PMC11178312; doi:10.1590/0034-7167-2023-0314)
Supplement: Translation

# Translation: Sentidos atribuídos à qualidade de vida relacionada à saúde pelas cuidadoras de adolescentes com diabetes

**DOI:** 10.1590/0034-7167-2023-0314pt

**Authors:** Marina Saraiva de Araújo Pessoa, Elisabeth Luisa Rodrigues Ramalho, Maria Elizabete de Amorim Silva Marinho, Elenice Maria Cecchetti Vaz, Lucila Castanheira Nascimento, Valéria de Cássia Sparapani, Neusa Collet

**Affiliations:** I Universidade Federal da Paraíba. João Pessoa, Paraíba, Brasil; II Faculdade de Goiana. Goiana, Pernambuco, Brasil; III Universidade de São Paulo. Ribeirão Preto, São Paulo, Brasil; IV Universidade Federal de Santa Catarina. Florianópolis, Santa Catarina, Brasil

**Keywords:** Diabetes Mellitus Tipo 1, Qualidade de Vida, Família, Adolescente, Cuidadores

## Abstract

**Objetivo:**

interpretar os sentidos atribuídos à qualidade de vida relacionada à saúde por cuidadoras de adolescentes com *diabetes mellitus* tipo 1.

**Métodos:**

estudo qualitativo, descritivo-exploratório desenvolvido no ambulatório de referência para doenças endócrinas em uma cidade da Paraíba com 14 cuidadoras de adolescentes com diabetes. As entrevistas ocorreram entre maio e setembro de 2021. O material empírico foi submetido à análise temática indutiva e interpretado à luz dos conceitos de qualidade de vida relacionada à saúde e do funcionamento familiar.

**Resultados:**

os sentidos atribuídos pelas cuidadoras à qualidade de vida relacionada à saúde dos adolescentes convergiram para sensação de estar com saúde, alimentação saudável, renda familiar satisfatória, envolvimento da família no cuidado e acesso efetivo à rede assistencial.

**Considerações Finais:**

o conhecimento destes sentidos possibilita que os profissionais de saúde elaborem estratégias que atendam às demandas singulares de cuidadores que vivenciam esse diagnóstico.

## INTRODUÇÃO

O número de crianças e adolescentes com *diabetes mellitus* tipo 1 (DM1) cresce anualmente, e grande parte deste público está no Brasil, que ocupa o terceiro lugar na faixa etária de zero a 19 anos com o diagnóstico da doença no mundo^(1)^. Como poucos países no mundo têm dados de incidência desta doença para menores de 19 anos, é provável a existência de dados subestimados^(2)^.

Na vivência dessa doença complexa, que exige contínuo gerenciamento de cuidados, muitos familiares se sentem despreparados, angustiados e culpados. Inicialmente, apresentam dificuldades na aceitação do diagnóstico, principalmente em relação à sua cronicidade^(3,4)^.

Os cuidadores geralmente fazem parte do núcleo familiar do adolescente com DM1, necessitando ser incluídos no plano de cuidados traçado para a família, uma vez que a qualidade de vida relacionada à saúde (QVRS) dos cuidadores também é afetada. Na maioria das vezes, as mães são as cuidadoras principais e vivenciam implicações biológicas, psicológicas e sociais desencadeadas pelas exigências de cuidados cotidianos com o adolescente com DM1. Assim, é necessário fortalecer a rede de apoio dessas famílias, buscando trazê-las para o foco da atenção à saúde^(5)^, facilitando a experiência com o DM1 e melhorando sua QVRS.

O conceito de “qualidade de vida relacionada à saúde” pode contribuir para compreender os sentidos da experiência vivida pelas famílias de adolescentes com DM1, por se tratar de uma construção subjetiva, multidimensional e dinâmica, associada a fatores individuais, sociais e ambientais, que proporcionam o bem-estar do indivíduo. O aspecto subjetivo desse conceito busca entender a percepção da pessoa sobre sua saúde e sua conjuntura de vida, a partir de como a doença interfere na vida do indivíduo^(6,7)^. Entender os sentidos atribuídos sobre a QVRS pelas cuidadoras de adolescentes com DM1 possibilita compreender quais circunstâncias influenciam suas QVRS, permitindo proporcionar aos familiares intervenções pautadas em suas necessidades.

A família e as cuidadoras desses adolescentes têm sido vistas como coadjuvantes no contexto da doença e na maioria das vezes, sofrem a maior sobrecarga no cuidado, interferindo diretamente no contexto biopsicossocial^(4)^. Diante das implicações para aqueles que convivem com os adolescentes com diabetes no núcleo familiar, levantou-se o seguinte questionamento: Quais os sentidos atribuídos pelas cuidadoras de adolescentes com DM1 sobre sua QVRS?

## OBJETIVO

Interpretar os sentidos atribuídos à qualidade de vida relacionada à saúde por cuidadoras de adolescentes com *diabetes mellitus* tipo 1.

## MÉTODOS

### Aspectos éticos

Este estudo seguiu os preceitos éticos do Conselho Nacional de Saúde e foi avaliado pelo Comitê de Ética em Pesquisa (CEP). Após a explicação e a compreensão do estudo por cada participante, o Termo de Consentimento Livre Esclarecido (TCLE) foi apresentado para assinatura e consentimento de ingresso na pesquisa. Nas entrevistas remotas, o TCLE foi enviado por meio de um *link* do Google Forms® utilizando o aplicativo WhatsApp. Para garantir a confidencialidade dos participantes, eles foram representados pela letra E de entrevistado, seguida de um numeral ordinal, que apresenta a ordem em que as entrevistas foram realizadas (E1, E2, ..., E14).

### Referencial teórico-metodológico

Adotou-se o Modelo Calgary de Avaliação da Família (MCAF)^(8)^, por permitir conhecer e planejar ações voltadas para as famílias. Esse modelo objetiva buscar soluções para minimizar danos emocionais, físicos e espirituais desencadeados pela doença na família^(8)^.

### Tipo de estudo

Pesquisa qualitativa, descritiva-exploratória, guiada pelas recomendações do *Consolidated Criteria for Reporting Qualitative Research* (COREQ)^(9)^. Optou-se por investigar a QVRS a partir da perspectiva qualitativa, devido às dimensões subjetivas desse construto, que relacionam o processo de saúde e doença com as repercussões na vida das pessoas, incluindo os aspectos físicos, mentais, sociais e ocupacionais^(10)^. Adicionalmente, o construto QVRS tem sido explorado na literatura por meio de instrumentos de mensuração, que certamente contribuem para sua compreensão, mas predeterminam sua avaliação a partir de um conjunto de itens e dimensões. A perspectiva qualitativa desse construto amplia a apreensão da QVRS, dando visibilidade às experiências das cuidadoras.

### Cenário do estudo

O estudo foi desenvolvido no Ambulatório de Endocrinologia de um hospital público de uma cidade de grande porte da Paraíba, na Região Nordeste do Brasil. O serviço é referência para o tratamento do DM1 no município estudado e em outros municípios do estado pactuados com a instituição.

### Coleta dos dados

Participaram do estudo cuidadores principais de adolescentes com DM1. A pessoa da família que cotidianamente realizava o gerenciamento do DM1 em parceria com o adolescente foi considerada como principal cuidador(a). Tal identificação se deu a partir da narrativa do membro da família abordado pela pesquisadora principal no primeiro contato, para exposição do objetivo do estudo e verificação dos critérios de seleção para ingresso na pesquisa.

Para o recrutamento dos participantes, percorreram-se etapas. Foi estabelecido contato presencial com os cuidadores no dia da consulta ambulatorial de acompanhamento dos adolescentes e também pelo aplicativo WhatsApp. Nesse último caso, o convite para participação foi feito individualmente a cada cuidador(a), por meio dos contatos do grupo de WhatsApp dos cuidadores das crianças e adolescentes com DM1 atendidos no ambulatório. A pesquisadora principal foi adicionada neste grupo após contato prévio com quem o administrava e também fazia seguimento no ambulatório em estudo. As aproximações e a coleta dos dados foram realizadas por uma enfermeira (primeira autora), que recebeu treinamento pelas demais integrantes da equipe, as quais têm ampla experiência em pesquisa, para a coleta dos dados qualitativos antes da entrada no campo de coleta.

Os critérios de inclusão adotados foram: ser o principal cuidador de adolescente com DM1 há pelo menos seis meses, pois é um período em que o cuidador apresenta um tempo de vivência com o tratamento; e ser a pessoa que realiza o gerenciamento diário do DM1 junto ao adolescente. Os critérios de exclusão foram: cuidadores de adolescentes que interromperam o acompanhamento há mais de seis meses, apesar de possuírem algum registro de atendimento no ambulatório; e aqueles sem condições e/ou habilidades tecnológicas para a entrevista remota.

As entrevistas foram realizadas entre maio e setembro de 2021, sendo dez delas na modalidade remota e quatro presenciais. O critério de suficiência fundamentou o encerramento das entrevistas, uma vez que o conjunto de dados coletados possibilitou a compreensão do objeto de estudo^(11)^.

### Análise dos dados

O material empírico foi submetido aos procedimentos para análise temática indutiva (ATI)^(12)^ e interpretado à luz do conceito de QVRS^(10)^ e do funcionamento familiar, conforme pressupostos do MCAF^(8)^. Nesse modelo, a categoria do funcionamento familiar é dividida em instrumental e expressiva. A instrumental encontra-se voltada para as Atividade da Vida Diária, por exemplo, fazer a aplicação de insulina, realizar as medidas de glicemia capilar, bem como outros aspectos do cuidado de adolescentes com DM1. Já o funcionamento familiar expressivo envolve nove outras categorias: comunicação emocional, comunicação verbal, comunicação não verbal, comunicação circular, solução de problemas, papéis, influência e poder, crenças, alianças e uniões^(8)^.

Na primeira fase da análise, a equipe de pesquisa realizou leituras repetidas das entrevistas transcritas na íntegra, para familiarização das pesquisadoras com o conjunto de dados e conhecimento de sua amplitude^(12)^. Esse conjunto de dados se constituiu no *corpus* da análise. Na segunda fase, iniciou-se o processo de geração dos códigos iniciais, possibilitando a caracterização inicial dos dados. Para isso, os extratos significativos foram destacados com cores diferentes, a partir de ferramenta do *Microsoft Office Word*®. Na terceira fase, os códigos foram agrupados de acordo com a afinidade e as relações estabelecidas entre eles para a geração de temas^(12)^.

Na quarta fase, após refinamento, os temas descritivos foram estabelecidos. Na quinta fase, foi construído um tema analítico a partir da síntese dos temas descritivos e articulados com os aspectos funcionais do MCAF. Na última fase, procedeu-se à análise reflexiva final, a partir do reconhecimento de que os extratos contemplavam a representação do objeto de estudo^(12)^, e da articulação entre os temas com a literatura e a interpretação das pesquisadoras.

Um conjunto de procedimentos garantiu o rigor desta pesquisa: foi desenvolvido um número suficiente de entrevistas com os participantes, o que permitiu aprofundar aspectos que colaboravam para ampliar a compreensão do objeto de estudo; os métodos e processos de coleta de dados foram descritos detalhadamente; apresentaram-se os dados de caracterização dos cuidadores para entender o contexto de vivência dos participantes; conduziu-se a análise minuciosa dos dados, com apresentação da interpretação dos resultados ilustrada com trechos extraídos dos relatos dos participantes e articulada com a literatura e aspectos funcionais do referencial teórico do MCAF^(8)^.

## RESULTADOS

Participaram do estudo 14 cuidadores de adolescentes com DM1; 13 mães e um pai com idades entre 34 e 56 anos. A maioria se autodeclarou com estado civil casado(a) e Ensino Médio completo. As ocupações relatadas com maior frequência foram agricultor e do lar. O tempo como cuidador(a) de um adolescente com DM1 variou entre três e 15 anos. Ressalta-se que dentre os 14 participantes, apenas um era do sexo masculino. Assim, como forma de normatização, será utilizado o termo cuidadora(s) ao longo do texto.

### Repercussões iniciais da doença para a cuidadora e a família do adolescente com DM1

Ao receber o diagnóstico de DM1 do filho, as cuidadoras vivenciam o sentimento de culpa, por acreditarem ter responsabilidade diante desse desfecho.

*Eu me senti como se fosse culpada de alguma coisa; achava que tinha sido por minha causa que ela tinha ficado assim.* (E10)

O modo como receberam a confirmação do diagnóstico do filho, nem sempre com uma comunicação compreensível, influenciou no enfrentamento inicial. Embora desnorteadas e sem compreenderem como seriam suas vidas daquele momento em diante, assumiram o papel de cuidadoras e tentaram ser fortes, para que o impacto do adoecimento sobre o filho fosse minimizado.

*Ela* [médica] *chegou e falou que ele* [filho] *estava diabético e que diabetes era uma doença que não existia cura. Ele já começou a chorar. Eu, como mãe, meu mundo ali desabou. Ele olhou para mim e perguntou: “mamãe, eu vou morrer?”. Eu disse: “vai não, meu filho”. Meu mundo caiu, mas em compensação, eu dei forças para ele seguir em frente.* (E2)

A carga de cuidados decorrente do tratamento inicial do DM1 pode gerar estresse para a cuidadora e toda a família. Em geral, por não conhecerem a doença e os cuidados necessários, a reação inicial foi de angústia, medo e negação. Ações proativas podem ser imobilizadas pela criação de linhas de fuga, dificultando o aprendizado e o enfrentamento dos cuidados cotidianos no domicílio.

*Ficamos muito angustiados, porque ninguém sabia cuidar. Meu esposo ficou aplicando* [insulina]*, mas chegou um dia que ele não estava em casa e eu tive que fazer, nervosa, tremendo. Eu tinha medo, só fazia chorar, dizia que não ia conseguir furar o dedo, que não iria conseguir aplicar* [insulina]. (E9)

### Qualidade de vida: novos sentidos a partir de reflexões entre o passado e o presente

Experienciar uma doença crônica como o DM1 na família influenciou na percepção das cuidadoras sobre sua QVRS, remetendo seu significado a fatores relacionados à saúde, à alimentação saudável, ao lazer, à atividade física, ao emprego, à união familiar, à ausência de doenças, à facilidade no acesso aos materiais e insumos, à tranquilidade e à paz. Para as participantes, qualidade de vida era:

[...] *a gente poder marcar um médico e ter mais espaço para fazer caminhada, academia. Ter uma boa alimentação, comer saudável, isso que é qualidade de vida.* (E1)

[...] *você viver em família, ter sua alimentação, ter saúde, sair um pouquinho, se distrair. É ter saúde, paz, tranquilidade*. [...] *é a pessoa ter seu emprego, ganhar seu dinheiro. Porque tendo o seu trabalho, não vai se humilhar pedindo a ninguém, vai ter como se alimentar bem com seus filhos, vai viver mais tranquila.* (E12)

Com o diagnóstico de DM1, houve reorganização das interações familiares e dos papéis a serem desempenhados. As cuidadoras passaram a ficar mais atentas e preocupadas com seus filhos e se sentiram menos livres em comparação ao período anterior ao diagnóstico. A QVRS foi considerada melhor quando não havia uma doença crônica na família.

*Eu me sentia mais livre, menos preocupada. Porque assim, se ele hoje não tivesse essa doença, por exemplo, e eu vou sair, não iria me preocupar o tanto que eu me preocupo em deixá-lo só.* (E13)

As cuidadoras renunciaram aos seus desejos, deixando de realizar atividades que faziam parte do seu dia a dia, como ir a lugares onde teria doces, para que seus filhos não se sentissem excluídos.

*Já deixei de ir para alguns cantos, por exemplo, aniversário que tem coisas doces, coisas gostosas, preferi não ir por causa dela, porque ir com ela e ela ficar só olhando o povo comendo, ficava chato.* (E5)

O DM1 também modificou a rotina do sono das cuidadoras, devido à preocupação com o manejo da doença ou pelas demandas de outros filhos. A possibilidade de ocorrer uma hipoglicemia noturna ou outras complicações desencadearam interrupções no sono e repouso.

*Impactou muito no meu sono, eu tinha que levantar várias vezes à noite, porque eu ficava preocupada se tinha baixado a glicemia, se ela* [filha] *estava respirando, ficava pensando mil coisas. No outro dia, amanhecia muito cansada porque não dormia à noite.* (E6)

A vida laboral das cuidadoras também ficou comprometida frente aos novos papéis a serem desempenhados no cuidado de seus filhos. Algumas tiveram que abandonar o emprego, pelas exigências diárias impostas pela terapêutica, fazendo com que se sentissem privadas pela atenção que precisavam oferecer aos filhos.

*Antes do diabetes dele eu saía, vendia um perfume, sempre fazendo algo, e depois do diabetes já foi uma coisa que me privou mais; a atenção era para ele, até porque no tempo* [do diagnóstico] *ele era tão pequeno, dependia de mim para aplicar insulina.* (E12)

### Seguir sozinha ou amparada: o apoio que faz a diferença no cotidiano

Foi difícil construir novas alianças para compartilhar o cuidado. Todavia, algumas cuidadoras relataram receber certas formas de apoio: dos seus cônjuges, na realização dos cuidados relacionados ao DM1 e no compartilhamento das obrigações do lar; de outros filhos, que ficavam atentos às complicações que os irmãos podiam apresentar; de outras mães, por meio de grupo em rede social, recebendo apoio informacional relacionado ao dia a dia com o DM1; de pessoas da sua religião/espiritualidade, com importante apoio para as cuidadoras “seguirem em frente”; e da referência ambulatorial, cujos profissionais eram vínculos fortes de apoio e confiança na assistência prestada desde o início do diagnóstico.

*Meu marido me ajuda e muito, quando precisa faz teste, aplica insulina, cuida também da dieta dele* [filho com DM1]. *Às vezes, eu preciso ir ao médico com uma das crianças, ele fica em casa e faz tudo, tanto em questão de afazeres de casa quanto com eles* [filhos]. *Minha filha que não tem DM1 presta muita atenção, às vezes eles estão sentindo alguma coisa e ela é muito atenta aos irmãos* [ambos com DM1]*, se eles estão precisando de alguma coisa, se estão passando mal. Ela é um grande apoio.* (E4)

*Eu me apeguei com Deus, pedi para Ele me ajudar. Eu teria que ser forte para ajudar, porque quem estava passando a situação difícil era ela* [filha com DM1] *e eu tenho certeza que Ele me ouviu, que a partir de então colocou paz no meu coração e entendimento para conseguir tocar a vida em frente.* (E6)

*Ajuda muito* [grupo de *WhatsApp*]*, tira dúvidas, como eu moro em uma cidade distante e quando faltavam algumas informações, não tinha como ligar para o C.* [local de dispensação de medicamentos] *para saber se tinha insulina, alguma coisa, as meninas do grupo sempre faziam isso. Sempre é assim, uma pessoa ajudando a outra ou com uma palavra.* (E12)

*Graças a Deus eu sou agradecida, porque eles* [profissionais do ambulatório] *são muito pacientes, estão sempre dispostos a fazer o que podem, a aconselhar, eu estou satisfeita. Eu tenho o contato de O. que é a enfermeira de lá e sempre que preciso de alguma informação, eu mando uma mensagem, e ela me retorna com a resposta.* (E14)

Um tipo de aliança importante nesse processo foi a estabelecida entre os amigos dos adolescentes com as cuidadoras e/ou com os próprios adolescentes, pois os amigos foram considerados como vínculos fortalecidos da rede social de apoio. O interesse dos amigos sobre o DM1 estimulou os adolescentes com diabetes a seguirem o tratamento corretamente, a cuidarem de sua saúde e a ficarem atentos a possíveis complicações agudas.

*Eles* [amigos da adolescente] *ficaram mais atenciosos, ligam para ela para saber como ela está, ligavam para mim para perguntar o que poderiam fazer. Eu expliquei a eles, disse que no dia que ela passar mal teriam que ligar para mim, para eu ir lá com a medicação. É bom dividir* [com os amigos dela]*, porque foi novo para todo mundo essa doença* (E3).

Mas nem todas as participantes receberam apoio familiar no manejo da doença no domicílio, dificultando a solução de problemas. Justificativas, como medo de aplicar a insulina e falta de coragem para realizar os cuidados, fizeram com que as cuidadoras se sentissem decepcionadas ou sozinhas para lidar com o diagnóstico e a terapêutica, fragilizando as relações intra-familiares e, consequentemente, as alianças e uniões entre seus membros. As pessoas do trabalho e os profissionais da escola do adolescente também foram indiferentes ao momento difícil vivido pelas cuidadoras e não ofertaram apoio.

*Ele* [pai do adolescente] *diz que não aplica a insulina porque tem medo. O irmão não se envolve porque não tem coragem, o cuidar é mais meu. No começo, eu me senti um pouco decepcionada, mas agora eu não ligo. Digo assim: “graças a Deus que me deu forças”.* (E2)

*Eu não recebi apoio no meu trabalho, de ninguém, acho que fizeram que não entendiam ou não queriam entender. E assim, na escola não era diferente, era muito complicado, porque professores não tinham conhecimento de como agir, do que fazer.* (E6)

### Novos impasses e ressignificações para o enfrentamento do diabetes

O tempo de experiência na gestão do DM1 influenciou positiva e negativamente a percepção da QVRS das cuidadoras no momento da entrevista. As que se adaptaram positivamente apresentaram melhor percepção da QVRS, enquanto as que apresentaram mais dificuldades em lidar com a doença relataram pior percepção sobre a QVRS. O primeiro caso esteve relaciona ao maior envolvimento da família nos cuidados, por exemp com a busca de uma vida saudável para todos os membros. no segundo caso, a sensação da falta de saúde, com a ren reduzida e o déficit no compartilhamento de responsabilidad levou a uma pior percepção.

*Eu acho que não é tão boa* [QVRS] *por conta que falta compreens falta diálogo, estou vendo que já tentei, conversei e cada vez fi mais difícil. Estou tentando melhorar minha qualidade de vid fazendo o que é melhor para mim, minha qualidade de sono. Est dormindo melhor porque eu não dormia, agora estou dormin a noite toda* [...]. (E10)

*Eu tenho a responsabilidade de pagar água, luz, telefone, internet para eles estudarem, e tudo isso sou eu sozinha, graças a Deus tem ajuda do B.* [auxílio governamental]*, que é o que vai ajudando também. Mas para dizer a você que a pessoa tem uma qualidade de vida sem um emprego e ganhar seu dinheiro, para pelo menos se alimentar bem, eu não considero qualidade de vida, não.* (E12)

Para o futuro, as cuidadoras demonstraram expectativas de mudanças positivas em sua saúde ao cuidarem do seu bem-estar. Já para o gerenciamento do cuidado, acreditaram na possibilidade de uma terapêutica oral, ao invés da insulina injetável, pelo incômodo relacionado à dor nas aplicações.

*Ultimamente, eu estou caminhando, comendo melhor, tentando levar uma vida saudável, porque eu estava acima do peso demais, eu acho que melhorei bastante.* (E10)

*Eu queria que fosse comprimido* [tratamento do DM1]*, não fosse insulina para não estar se furando. Me incomoda, eu acho que para mim, dói.* (E7)

Também gostariam que existissem pessoas para compartilhar o cuidado ao filho com DM1 no cotidiano, fortalecendo alianças e para que pudessem se desvincular um pouco da função de cuidadora.

*Gostaria de ter aquela pessoa presente que ajudasse, mas é eu ou ele* [filho com DM1]*, se eu for obrigada a sair, ele* [filho DM1] *se cuida, aplica* [insulina] *e já come o que eu deixei.* (E9)

### Trilha da qualidade de vida relacionada à saúde de cuidadoras de adolescentes com DM1

A Figura 1 representa o tema analítico “Trilha da qualidade de vida relacionada à saúde de cuidadoras de adolescentes com DM1: transições no gerenciamento do cuidado e mobilização para o resgate da homeostase familiar”. O termo “trilha” foi utilizado para representar metaforicamente os caminhos percorridos na busca para a solução dos problemas e para as dificuldades enfrentadas pelas cuidadoras na reconstrução da homeostase familiar e da QVRS. Entretanto, cada cuidadora apresentou singularidades no modo de enxergar o futuro de si, da família e do adolescente com DM1, influenciadas pelo modo como as dimensões instrumentais e expressivas do funcionamento familiar eram estabelecidas antes, durante e após esse diagnóstico.

**Figura 1:** Trilha da qualidade de vida relacionada à saúde de cuidadoras de adolescentes com DM1, 2022

As cuidadoras enfrentaram diferentes vivências cotidianas a partir do diagnóstico de um filho com DM1. Perceberam a diminuição da QVRS devido à grande demanda de cuidados, restrições, sentimentos negativos e incertezas, que passaram a fazer parte da vida familiar.

Após um tempo significativo de experiência, a percepção das cuidadoras sobre a QVRS foi influenciada pelo domínio do conhecimento sobre DM1, relações familiares, rede de apoio e adesão e comprometimento do adolescente ao tratamento. Tal percepção foi maior nas famílias que compartilharam o cuidado entre si e foram acompanhadas pelo serviço de referência ambulatorial. Desse modo, as cuidadoras percorreram constantemente uma trilha retroativa e sem fim.

O modo como enfrentaram a doença foi influenciado por relações familiares preexistentes ao DM1; barreiras relacionadas aos insumos necessários; assistência à saúde local que receberam; dificuldades na adaptação familiar após o diagnóstico e novos impasses que podiam surgir ao longo do tempo. As interações familiares e a mobilização de todos os membros da família foram importantes para que as mães/cuidadoras se sentissem acolhidas e menos sobrecarregadas diante do que estavam vivenciando. A união familiar no desempenho de diferentes papéis interferiu diretamente na percepção da QVRS das cuidadoras. Quando a família permaneceu ou se tornou unida, houve maior percepção de tranquilidade e paz por parte da cuidadora, assim como disponibilidade de tempo para realizar atividade física e ter um emprego formal e momentos de lazer.

## DISCUSSÃO

Dentre os 14 cuidadores de adolescentes com DM1 participantes desta pesquisa, 13 foram mães e apenas um pai. Isso permite tecer considerações acerca do funcionamento expressivo familiar no aspecto crenças. As mulheres assumem esse lugar de cuidado na família, pois são consideradas responsáveis pela saúde dos filhos^(13)^. As diferenças de gênero colocam mais frequentemente as mães no papel de cuidadora, o que pode impactar negativamente no funcionamento familiar^(8)^.

As cuidadoras tiveram experiências difíceis diante do diagnóstico do DM1. A aceitação de um filho com uma doença crônica é um processo contínuo, no qual os sentimentos iniciais podem ser transformados em conformação à medida que os adolescentes seguem o tratamento^(14)^. O acolhimento das mães e pais pelos profissionais e a forma como as informações são compartilhadas com eles afetam o entendimento sobre a doença e podem minimizar ou maximizar o sofrimento inicial das cuidadoras. É importante que os profissionais sejam empáticos e sensíveis, buscando compreender o impacto psicológico e emocional sofrido pelas cuidadoras e pelos adolescentes. Uma abordagem sensível e humanizada viabiliza a formação de vínculos estreitos entre os profissionais e a família, oportunizando o estabelecimento de planos de cuidados mais eficazes^(15)^.

Para minimizar ansiedades e preocupações, as cuidadoras necessitam da parceria e do auxílio de outros membros da família. Assim, podem se sentir seguras em compartilhar seus receios^(16)^. A relutância em aprender as práticas de cuidado pode refletir no modo como os filhos enfrentam o diagnóstico. Portanto, os profissionais da saúde também exercem papel fundamental na orientação para o cuidado^(17)^.

Cuidar de um adolescente com DM1 envolve fatores econômicos, afetivos, sociais, comportamentais, psicológicos e profissionais. Portanto, é um processo dinâmico e complexo que também depende de qualidades pessoais e de habilidades do cuidador^(5)^. Os aspectos trazidos pelas cuidadoras sobre sua QVRS vão ao encontro de seu conceito, pois mudanças no estado de saúde de um filho influenciam o estilo de vida e a satisfação social da família, que são determinantes no enfrentamento da doença^(10)^. Adicionalmente, os vínculos entre os membros da família ou laços emocionais antes do diagnóstico de DM1 influenciam na união familiar frente ao processo de saúde-doença. Quando os laços familiares são estreitos previamente ao diagnóstico, a inserção de todos membros da família no cuidado é mais fácil^(8)^.

De modo geral, estudo indica que a qualidade de vida de cuidadoras de crianças e adolescentes com alguma doença crônica foi influenciada negativamente pela quantidade de filhos com essa condição, pelo uso de medicações pela cuidadora e por limitações financeiras. Já os fatores de proteção, que favorecem a qualidade de vida, foram ter casa própria e o apoio recebido^(18)^.

Experiências iniciais negativas também foram identificadas em estudo que considerou o período logo após o diagnóstico como um momento de disrupção na vida das cuidadoras. Esse rompimento foi relacionado ao comprometimento das atividades domésticas, sociais e profissionais, evidenciando dificuldades na solução de problemas no sistema familiar. Dificuldades de aceitação das mudanças também foram evidenciadas na nova rotina imposta. Por isso, é prioritário que o suporte de uma equipe multiprofissional esteja acessível de maneira constante, a fim de minimizar preocupações e angústias que afetam negativamente a QVRS^(19)^.

O DM1 no seio familiar e as adversidades decorrentes da doença criam novas relações entre os membros da família. Apoiadas no MCAF, pode-se afirmar que tais relações podem ser positivas, quando possibilitam que o cuidado ao adolescente seja compartilhado com outros membros, ou negativas, quando a mãe assume todas as responsabilidades^(8)^. As relações são influenciadas pela idade da criança/adolescente; pelo equilíbrio emocional paterno diante do diagnóstico, comumente maior comparado ao das mães, visto que na comunicação emocional. os pais são mais difíceis de expressar afetuosidade; e, pelos vínculos que geralmente são fortalecidos entre mãe e filho, diferentemente do pai, que pode ser excluído dos cuidados pelas cuidadoras, quando não houver acompanhamento psicológico e orientações^(20)^.

Diante da realidade apresentada, compreende-se que os significados atribuídos sobre QVRS pelas cuidadoras também estão relacionados ao funcionamento de cada família. Nas situações em que o adolescente exige grande demanda de cuidados, principalmente no início do diagnóstico, as cuidadoras podem se sentir esgotadas ou frustradas pelo não desempenho de suas atividades individuais. Dessa forma, os sentidos da QVRS das cuidadoras são influenciados tanto pelo modo como apreendem o funcionamento familiar, quanto pelo modo como compartilham (ou não) os cuidados entre os membros. O estabelecimento de novas rotinas relacionadas aos aspectos instrumentais do MCAF como uma resposta comportamental ativa à doença, com participação de todos os membros família, são mecanismos importantes para manutenção do senso de normalidade familiar^(8)^.

Na dimensão expressiva do funcionamento familiar, as cuidadoras assumem o papel central no cuidado e passam a ter o sono interrompido para atender às demandas do filho com DM1. As preocupações com complicações agudas desencadeadas durante o sono do filho geram ansiedade e estresse, impactando no repouso delas. Uma vez que o sono prejudicado tem implicações psicossociais na vida das cuidadoras, é fundamental avaliar o sono da família e dos adolescentes. O gerenciamento noturno pode ser melhor adaptado, instituindo-se o compartilhamento do cuidado noturno pelos diferentes membros da família, quando possível^(21)^.

Para as cuidadoras, estar inseridas na vida laboral contribui para uma melhor QVRS. Porém, as demandas cotidianas no gerenciamento do cuidado com o filho as privam dessa opção. Assim, a renda familiar, com frequência, fica comprometida, o que, associado à baixa escolaridade, leva a família à situação de vulnerabilidade. O comprometimento da renda pode dobrar as chances de ser baixa a QV das cuidadoras de uma criança ou adolescente com doença crônica^(18)^. Além disso, todos os membros da família passam a depender financeiramente de um único membro – neste caso, do pai. Tal fato associa-se negativamente aos papéis no compartilhamento do cuidado.

Observa-se, frequentemente, que a renda do marido se relaciona negativamente à divisão de papéis na família, enquanto a educação da esposa se relaciona positivamente. De acordo com o MCAF, a figura paterna encontra-se fortemente associada aos aspectos socioeconômicos da família e menos ativamente ao seu funcionamento, diferente das mulheres. Os pais têm padrões mais rígidos e dificuldade de expressar emoções, o que interfere na comunicação emocional da família^(8)^.

A rede de apoio é um dos mais importantes influenciadores positivos para a família e os adolescentes. A própria família, a rede social e a religião são capazes de contribuir para superar as dificuldades e a sobrecarga enfrentadas pelas cuidadoras após o diagnóstico. A família auxilia com afeto, suporte financeiro e ações no dia a dia; os grupos das mídias sociais ajudam na falta de insumos e pelas trocas de experiências; e a religião, em aspectos emocionais de conforto^(8,22)^. As crenças religiosas ou espirituais são importantes para os aspectos funcionais da família, pois estão associadas aos seus valores e à cultura. Quando algum membro sofre um problema de saúde, as crenças restritas podem dificultar o enfrentamento da doença e gerar sofrimento pela falta de perspectiva^(8)^.

A equipe de educação em diabetes do serviço ambulatorial de referência se torna importante fonte de apoio para a família, principalmente durante a fase de adaptação às demandas da doença. Os profissionais transmitem às cuidadoras maior confiança e segurança nas orientações que recebem, reduzindo barreiras entre a assistência e as famílias, melhorando o controle da doença a partir de motivações geradas nos adolescentes. A acessibilidade a uma equipe de profissionais que buscam entender a realidade familiar e planejam a assistência de acordo com suas necessidades proporciona maior satisfação com a atenção em saúde recebida^(23)^. O MCAF é uma importante ferramenta que pode ser utilizada pelos profissionais para macroavalição das famílias, visto que o planejamento de intervenções direcionadas para a resolução de problemas pode interferir positivamente no funcionamento familiar.

O suporte dos amigos do adolescente traz segurança para as mães, principalmente na escola ou em momentos de interação social. Para os adolescentes, pode ser fundamental para a aceitação da doença e a adesão ao tratamento quando os amigos se preocupam com o bem-estar deles e têm interesse em aprender sobre o diabetes, podendo ajudá-los em casos de identificação e correção das alterações glicêmicas^(24)^.

O suporte social oferecido pelos pares também se torna importante no processo de enfrentamento, por meio de troca de experiências e informações, e por facilitar a aquisição de materiais e insumos para a terapêutica, por exemplo. Além disso, contribui para a elevação da estima por meio dos relacionamentos estabelecidos. Tais ações fortalecem comportamentos adaptativos e a autoconfiança e proporcionam visibilidade para as cuidadoras^(25)^.

Corroborando os achados, a falta de apoio da escola do adolescente também ficou evidente em estudo realizado na Arábia Saudita, que buscou investigar a segurança das escolas e as diferenças no tratamento do diabetes entre escolas públicas e privadas. Os pais tiveram má percepção dos cuidados realizados, pois as crianças e os adolescentes se responsabilizavam sozinhos pelo tratamento, principalmente nas escolas públicas^(26)^. Em estudo brasileiro, a equipe escolar apresentou baixo conhecimento e dificuldades para compreender as necessidades de crianças e adolescentes com DM1, ressaltando a necessidade de políticas públicas voltadas para a educação sobre o manejo adequado da doença nas escolas^(27)^, o que é fundamental, visto tratar-se de indivíduos em díade escolar.

Observa-se que relações de gênero, representadas pelas mães assumindo as responsabilidades pelo cuidado, marcam a definição dos papéis, e, com frequência, a sobrecarga de tarefas leva a prejuízos na saúde mental e física das cuidadoras^(8,28)^, com diminuição da QVRS em comparação com a população geral. Em contrapartida, elas utilizam a resiliência para lidar com a sobrecarga de cuidados, com visão diferente sobre o que estão vivenciando e foco na resolução dos problemas^(8)^. Esses mecanismos de enfrentamento ajudam a desenvolver habilidades para suportar as dificuldades impostas pelo DM1, assim como a se concentrar em sua capacidade de responder de forma positiva às demandas cotidianas do filho.

Apesar de os filhos com DM1 crescerem e começarem a desenvolver autonomia no cuidado de si, a carga de preocupações continua elevada, prejudicando a saúde das cuidadoras pelo estresse físico e mental, que é potencializado por relações familiares desgastantes. Um plano de cuidados com a finalidade de reduzir conflitos, que envolva a família no processo, tende a melhorar a percepção das cuidadoras sobre sua saúde^(29)^.

Estudo brasileiro sobre a QV de cuidadoras de crianças e adolescentes com DM1 apontou baixas pontuações nos domínios físico, psicológico e ambiental. Esses aspectos refletem baixos sentimentos positivos, na autoestima, na imagem corporal, nos fatores socioeconômicos e no sono, levando a prejuízos nas condições de saúde^(30)^. Já outro estudo com famílias libanesas, no qual a maioria das mães não possuía nível superior e era dona de casa, mostrou que a percepção da QVRS familiar estava diretamente ligada a problemas funcionais do grupo familiar^(31)^. É importante conhecer como eram estabelecidas as relações pré-diagnóstico do DM1 e a cultura familiar, além das relações familiares atuais, pois esses fatores interferem na melhor ou pior adaptação da família à doença^(8)^.

A QVRS das cuidadoras deste estudo está relacionada às mudanças em aspectos sociais e financeiros que os cuidados com o adolescente desencadeiam no seio familiar. As experiências vivenciadas por elas após um tempo de diagnóstico melhoraram a visão sobre o DM1, apesar do cotidiano ainda ser permeado por preocupações que muitas vezes levam a desentendimentos no núcleo familiar. A QVRS dessas cuidadoras é influenciada por vários determinantes, e o envolvimento da família é um dos mais importantes. Porém, isso dependeu de como esses laços foram formados entre os membros e da corresponsabilidade ou não de cada um pelo cuidado do adolescente com DM1.

As cuidadoras possuem expectativas otimistas, nas quais projetam melhorias futuras para suas condições de saúde e as de seus filhos, bem como crenças de cura, que sinalizam a necessidade de diminuir sentimentos negativos^(32)^, o que pode levar a futuras frustrações. Apesar das dificuldades encontradas pelas cuidadoras no dia a dia do enfrentamento do DM1, as expectativas podem gerar motivações para que elas cuidem de seu próprio bem-estar.

A Figura 1 “Trilha da qualidade de vida relacionada à saúde de cuidadoras de adolescentes com DM1: transições no gerenciamento do cuidado e mobilização para resgate da homeostase familiar” representa o dia a dia das famílias entrevistadas neste estudo. Com o diagnóstico do DM1 dos filhos, houve modificações dos aspectos instrumentais e expressivos do funcionamento familiar, em especial da cuidadora. Famílias mais flexíveis a adaptações, que compartilham as responsabilidades de cuidado com o adolescente e suas preocupações tendem a uma coalizão positiva. Acompanhar essas famílias se tornou primordial, pois os profissionais de saúde podem ajudá-las a encontrar sua homeostase, favorecendo o funcionamento familiar.

### Limitações do estudo

Como limitação do estudo, destaca-se a participação de um único pai cuidador, impossibilitando diferenciar e aprofundar as singularidades da figura do pai no cuidado com filho com DM1.

### Contribuições para a área da saúde

Para a prática, as implicações deste estudo podem contribuir na compreensão subjetiva da QVRS das cuidadoras e famílias, possibilitando que políticas públicas e práticas profissionais sejam criadas e/ou aperfeiçoadas, a fim de garantir um cuidado singular, integral e efetivo, para melhorar a QVRS das cuidadoras, famílias e adolescentes com DM1. Assim, estarão fortalecendo-os e auxiliando-os a encontrar suas próprias soluções frente a adversidades do cotidiano.

## CONSIDERAÇÕES FINAIS

A percepção das cuidadoras entrevistadas sobre os sentidos atribuídos à QVRS tem relação com a percepção de saúde, bem-estar e afastamento de doenças; o acesso à alimentação saudável por toda família; os momentos de divertimento coletivo; as práticas de exercícios físicos sem restrições; o emprego formal como uma fonte de renda segura; o viver com serenidade; a manutenção da união familiar; e com a obtenção sem burocracia dos insumos necessários aos cuidados, com o acesso à rede assistencial de saúde. No geral, a QVRS foi considerada pior pelas cuidadoras no início do diagnóstico de DM1 dos(as) filhos(as), mas apresentou melhora no transcorrer do tempo de experiência das famílias. A grande maioria ainda não a considera como satisfatória, apesar das inúmeras vivências relacionadas à doença.

Já a percepção otimista do funcionamento familiar estava presente nas famílias que apresentaram rede de apoio formada pelos cônjuges e por outros filhos que ajudavam nas demandas de cuidado ao DM1 e nas atividades domésticas; pelos próprios adolescentes comprometidos com o seu tratamento; pelos amigos dos(as) filhos(as), estimulando-os(as) a seguir o tratamento; pelas outras famílias que vivenciam a mesma condição e pelas mídias sociais, que proporcionaram informações e doações de insumos; e pelo acolhimento dos profissionais do ambulatório. Assim, com o suporte dado, mudanças negativas relacionadas ao DM1 tiveram menor impacto na vida das cuidadoras.
